# PROTOCOL: Behavioral, information and monetary interventions to reduce energy consumption in households: A “living” systematic review

**DOI:** 10.1002/cl2.1424

**Published:** 2024-07-10

**Authors:** Tarun M. Khanna, Diana Danilenko, Mark Andor, Max Callaghan, Julian H. Elliott, Tim Repke, Luke A. Smith, Jorge Sanchez, T. V. Bhumika, Jan C. Minx

**Affiliations:** ^1^ University of British Columbia Vancouver Canada; ^2^ MCC‐Berlin Berlin Germany; ^3^ RWI Essen Germany; ^4^ Monash University Melbourne Australia; ^5^ Campbell South Asia New Delhi India

## Abstract

This is the protocol for a Campbell systematic review. The objectives are as follows: Our proposed systematic review and meta‐analysis will integrate the evidence available from all sources to answer the following questions: (1) to what extent can information, behavioral and monetary interventions reduce energy consumption of households in residential buildings? (average treatment effect of interventions) (2) what is the relative effectiveness of interventions? (account for heterogeneity in treatment effects across and within studies) (3) how effective are combinations of different interventions?

## 1 BACKGROUND

### 1.1 The problem, condition, or issue

Policymakers only have little time left to prevent the worst impacts of climate change and limit global warming to well below two degrees (IPCC, 2022, 2023). Assessments by the Intergovernmental Panel on Climate Change (IPCC) have provided evidence about the extent of climate change and possible pathways for mitigation (IPCC, 2023). However, rigorous solution‐oriented knowledge on how to facilitate those pathways is missing (Berrang‐Ford et al., 2020; Minx et al., 2017). In particular, a systematic assessment of the available scientific evidence of is not available to understand what climate policies work, to what extent, in what context, when and for whom (Berrang‐Ford et al., 2020). This living systematic review will demonstrate how scientific evidence on the effectiveness of one particular set of policy interventions, namely behavioral, information and monetary interventions in household energy demand, can be kept up‐to‐date to deliver rigorous solution‐oriented knowledge to policymakers whenever they need it.

Finding low energy demand pathways is necessary to hedge against the risks involved in decarbonizing energy supply and is key for finding socially acceptable ways of meeting the Paris climate goals (Creutzig et al., 2022; Grubler et al., 2018; Van Vuuren et al., 2018). There has been a surge in interest in using demand‐side policies, particularly aimed at behavioral change, for reducing energy demand and the consequent emissions. To reduce energy demand of households, primary studies have experimented with using monetary incentives and non‐monetary interventions, including nudging, appealing to norms, and providing easily interpretable and credible information at the point of decision‐making. The relevant literature is spread across various disciplinary fields: economics, psychology, power system, engineering studies. Our proposed review and meta‐analysis will integrate the evidence available from all sources to compare the effectiveness of different types of interventions (sometimes deployed simultaneously) and to understand what drives the variation in outcomes across studies.

### 1.2 The intervention

We will perform a systematic review and meta‐analysis of the literature on interventions in residential energy demand to reduce energy usage. These interventions include monetary incentives that offer households a tangible financial reward for reducing energy consumption, behavioral interventions like nudging, appealing to norms, and providing easily interpretable and credible information at the point of decision‐making as well as improving skills required to perform or forego behaviors. A detailed typology of the interventions along with a brief description of each type of intervention is given in Table 1.

**Table 1:** Detailed typology of interventions.

Intervention type		Intervention	Description
Information and education	Information	Tips Energy Audit Normative messaging	Information policies essentially attempt to address and behavioral and information asymmetries. They include a transfer of information to consumers and households like in the form of energy audits.
Behavior‐based interventions	Feedback	Home displays Historical Redesign utility bills Smart Meters	These interventions show past behavior of consumers to make them aware of their usage patterns. Like the use of smart meters which show visual information on use of appliances or home displays or sharing historical use reports. They could also include the redesigning of utility bills that show past information.
	Motivation	Commitment Devices Goal Setting Gamification	Social pressure has also been employed in the form of public pledges or commitments by households to practice energy conserving behaviors. Goal setting interventions in which households commit to reducing energy consumption by a certain percentage over the course of the experiment are other commitment devices. Some recent experiments have used web based gamified platforms or mobile apps to induce behavioral change.
	Social Comparison	Home energy reports Normative feedback	Households are benchmarked against the performance of their social group. Norm based communication has been widely adopted by utilities in the form of Home Energy Reports, which seem to be effective in some cases even years after households received their initial reports.
Monetary	Rewards/Rebates/Pricing	Rewards, rebates and time of use pricing/critical peak pricing	Time of use pricing aligns the prices faced by households with the underlying cost of supply, which is higher during peak demand periods. Other interventions reward consumers for reducing peak period consumption.

### 1.3 How the intervention might work

There is a rich theoretical literature into possible pathways that drive reduction in household energy consumption in response monetary, information or behavior based interventions. We do not aspire to provide a comprehensive review of the theory of change for each intervention. See Abrahamse and Steg (2013), Andor and Fels (2018), Fischer (2008), Hahn and Metcalfe (2016) for a detailed theoretical discussion.

### 1.4 Why it is important to do this review

There is a well‐established and fast‐growing literature on monetary and non‐monetary interventions in energy consumption in households, including nudging, appealing to norms, providing easily interpretable and credible information at the point of decision‐making, and improving skills required to perform or forego behaviors. The relevant literature is spread across various disciplinary fields: economics, psychology, power system studies.

Previous meta‐analyses on this topic are intra‐disciplinary (e.g., Abrahamse et al., 2005) and/or focused on a subset of interventions. For instance, Faruqui and Sergici (2013) focus on pricing interventions, Karlin et al. (2015) on feedback and Andor and Fels (2018) on social comparison, commitment devices, goal setting, and labeling. Nisa et al. (2019) consider evidence from a wider range of household behaviors that are relevant for climate change mitigation but do not review interventions in energy consumption exhaustively. The meta‐analysis by Delmas et al. (2013) was based on a narrower literature search and did not include studies published after 2012. A review with similar scope but with studies updated till 2019 was published by Buckley (2020). Khanna et al. (2021) provides a comprehensive meta‐analysis but the literature on behavioral interventions is increasing fast, so reviews need to be constantly updated.

The latest systematic review by Khanna et al. (2021) contained 360 effect sizes from 122 studies with evidence from 25 different countries published by mid‐2020. Since then, considerably more evidence has emerged in this fast‐growing field of study as technological advancement in metering of energy and information provision has made it easier to experiment with such interventions. The new knowledge gained from these studies may lead to changes in evidence‐based recommendations for policymakers and practitioners. However, there are long lags in incorporating new evidence. To prevent such gaps in knowledge, this review will systematically and continually update the evidence using the “living systematic review” concept, hitherto mainly discussed in clinical sciences (Akl et al., 2017; Elliott et al., 2017; Simmonds et al., 2017; Thomas et al., 2017). This living systematic review will provide up‐to‐date evidence on the efficacy of such interventions while simultaneously preventing duplication of effort in incorporating past studies.

Methodologically, this review filles several gaps also from a methodological perspective: understanding the role of machine learning (ML) in updating reviews, resolving the statistical challenges in updating meta‐analysis, and setting up the guidelines for updating policy recommendations based on a living review.

## 2 OBJECTIVES

Our proposed systematic review and meta‐analysis will integrate the evidence available from all sources to answer the following questions: (1) to what extent can information, behavioral and monetary interventions reduce energy consumption of households in residential buildings? (average treatment effect of interventions) (2) what is the relative effectiveness of interventions? (account for heterogeneity in treatment effects across and within studies) (3) how effective are combinations of different interventions?

## 3 METHODS

We start this study by replicating the methods from Khanna et al. (2021) as it provides a rigorous and comprehensive evidence base and serves a suitable case for living evidence. We therefore adopt all the definitions and conventions from this study and add the elements required to turn it into a living systematic review and meta‐analysis. We will adhere to the MECCIR reporting standards and fill out the AMSTAR2 checklist along with the review. The relevant portions of the MECCIR standards for protocol have been filled in and are attached.

### 3.1 Criteria for considering studies for this review

#### 3.1.1 Types of studies

We will include studies that conduct randomized control trials to estimate the effect of the relevant interventions. We also include studies with quasi‐experimental that estimate a causal effect including difference‐in‐difference, IV, longitudinal studies, and so forth. We do not include studies that are purely theoretical or simulate the effect of studies using constructed data.

#### 3.1.2 Types of participants

We will include all studies that estimate the effect at the household level, or common living spaces like dormitories.

#### 3.1.3 Types of interventions

We will perform a systematic review and meta‐analysis of the literature on interventions in residential energy demand to reduce energy usage. These interventions include monetary incentives that offer households a tangible financial reward for reducing energy consumption, behavioral interventions like nudging, appealing to norms, and providing easily interpretable and credible information at the point of decision‐making as well as improving skills required to perform or forego behaviors. Following Khanna et al. (2021), we classify the studied interventions into five categories—monetary incentives, information, feedback, social norms and motivation interventions. Refer to Table 1 for details about each type of interventions.

#### 3.1.4 Types of outcome measures

##### Primary outcomes

Energy or electricity consumption of the household.

##### Secondary outcomes

N/A.

##### Duration of follow‐up

We will separately code the duration of the baseline period, duration of the intervention and duration of the follow‐up. However, it should be noted that from our understanding of the literature not many studies do follow‐ups.

##### Types of settings

We will include all experimental and quasi‐experimental studies conducted at the household level. We will only include studies that capture actual energy consumption behavior so we will exclude simulation studies or studies conducted in a laboratory setting that only capture intent to save. This strategy is considered optimal as there is already a large literature that captures experiments in the real world such that evidence from online or lab experiments is not needed to be included.

### 3.2 Search methods for identification of studies

The starting point of our search would be the studies identified by Khanna et al. (2021) through their comprehensive search of the relevant literature in previous systematic reviews and meta‐analyses, and bibliographic databases. Khanna et al. (2021) used a prioritized screening approach (Callaghan & Müller‐Hansen, 2020) to identify relevant evidence from over 64,000 studies at the abstract level (through a mix of manual and ML approaches), whereby 934 studies were screened manually at the full‐text level and 122 relevant studies were identified, which are twice as many as identified by any previous systematic review with this scope and includes all the studies identified by previous reviews. We will update this pool of relevant studies through string‐based searches of bibliographic databases and gray literature since 2020, when the databases were searched by Khanna et al. (2021). The search string to be used will follow the PICOS (population, intervention, comparator, outcome, and study design) logic to target empirical studies covering one or more of such interventions and household energy consumption as the outcome variable (Table 2).

**Table 2:** Search string used in Web of Science/Scopus/Medline advanced search.

Database	Population	Intervention	Outcome
Scopus	((household* OR residential OR building OR dormitor* OR individual OR consumer* OR participant* OR customer* OR domestic OR homeowner*)	(feedback OR pric* OR {time‐of‐use} OR {time‐of‐day} OR {real time} OR {peak} OR {dynamic pricing} OR “smart meter*” OR “smart grid*” OR (behavioral AND (economic* OR intervention* OR guideline*)) OR nudge* OR {choice architecture} OR norm OR norms or {normative} OR {social influence} OR {block leader} OR {public commitment} OR {social comparison} OR {social learning} OR {social modeling} OR {peer comparison} OR {peer information} OR salience OR “commitment device*” OR {Pre‐commitment} OR {precommitment} OR pledge OR {behavioral contract} OR {commitment contract} OR “commitment approach*” OR {personal commitment} OR audit OR rebate OR reward OR incentives OR {goal setting} OR {home energy report} OR {in‐home display} OR (information W/3 (campaign* OR provision OR strategies OR acquisition OR intervention* OR system*)) OR {foot‐in‐the‐door} OR {minimal justification} OR “applied game*” OR “serious game*” OR gamif* OR {dissonance} OR tariff OR “time‐varying pricing”)	(((energy OR electric* OR gas) W/15 (consumption OR conservation OR efficiency OR use OR demand OR usage)) OR “price responsiveness”))
Web of Science	((household* OR residential OR building OR dormitor* OR individual OR consumer* OR participant* OR customer* OR domestic OR homeowner*)	(feedback OR pric* OR “time‐of‐use” OR “time‐of‐day” OR “real time” OR “peak” OR “dynamic pricing” OR “smart meter*” OR “smart grid*” OR (behavioral AND (economic* OR intervention* OR guideline*)) OR nudge* OR “choice architecture” OR norm OR norms or “normative” OR “social influence” OR “block leader” OR “public commitment” OR “social comparison” OR “social learning” OR “social modeling” OR “peer comparison” OR “peer information” OR salience OR “commitment device*” OR “Pre‐commitment” OR “precommitment” OR pledge OR “behavioral contract” OR “commitment contract” OR “commitment devices” OR “commitment approach*” OR “personal commitment” OR audit OR rebate OR reward OR incentives OR “goal setting” OR “home energy report” OR “in‐home display” OR (information NEAR/3 (campaign* OR provision OR strategies OR acquisition OR intervention* OR system*)) OR “foot‐in‐the‐door” OR “minimal justification” OR “applied game*” OR “serious game*” OR gamif* OR “dissonance” OR “goal setting” OR tariff OR “time‐varying pricing”)	(((energy OR electric* OR gas) NEAR (consumption OR conservation OR efficiency OR use OR demand OR usage)) OR “price responsiveness”))
JSTOR (through Constelate)	(household* OR residential OR building OR dormitor* OR individual OR consumer* OR participant* OR customer* OR domestic OR homeowner*)	(feedback OR pric* OR “time‐of‐use” OR “time‐of‐day” OR “real time” OR “peak” OR “dynamic pricing” OR “smart meter*” OR “smart grid*” OR (behavioral AND (economic* OR intervention* OR guideline*)) OR nudge* OR “choice architecture” OR norm OR norms or “normative” OR “social influence” OR “block leader” OR “public commitment” OR “social comparison” OR “social learning” OR “social modeling” OR “peer comparison” OR “peer information” OR salience OR “commitment device*” OR “Pre‐commitment” OR “precommitment” OR pledge OR “behavioral contract” OR “commitment contract” OR “commitment devices” OR “commitment approach*” OR “personal commitment” OR audit OR rebate OR reward OR incentives OR “goal setting” OR “home energy report” OR “in‐home display” OR “information campaign”~3 OR “information provision”~3 OR “information strategies”~3 OR “information acquisition”~3 OR “information intervention”~3 OR “information system*“~3 OR “foot‐in‐the‐door” OR “minimal justification” OR “applied game*” OR “serious game*” OR gamif* OR “dissonance” OR “goal setting” OR tariff OR “time‐varying pricing”)	(“energy consumption”~15 OR “electric consumption”~15 OR “electricity consumption”~15 OR “gas consumption”~15 OR “energy conservation”~15 OR “electric conservation”~15 OR “electricity conservation”~15 OR “gas conservation”~15 OR “energy efficiency”~15 OR “electric efficiency”~15 OR “electricity efficiency”~15 OR “gas efficiency”~15 OR “energy use”~15 OR “electric use”~15 OR “electricity use”~15 OR “gas use”~15 OR “energy demand”~15 OR “electric demand”~15 OR “electricity demand”~15 OR “gas demand”~15 OR “energy usage”~15 OR “electric usage”~15 OR “electricity usage”~15 OR “gas usage”~15 OR “price responsiveness”~15)
Google Scholar	(household* OR residential)	(information OR feedback OR price OR incentives)	(“electricity consumption” OR “energy consumption” or “energy conservation”)

### 3.3 Electronic searches

We will search all the relevant databases: Web of Science Core Collections Citation Indexes (the topic field, which includes title, abstract, author keywords and keywords plus), Scopus (title, abstract, keywords), JSTOR (title, abstract), RePec (title and keywords) and the web‐based academic search engine Google Scholar (title) and Policy Commons (title, summary). For Google Scholar, we use Publish or Perish to download the relevant search results. We split the query by intervention type, implement partial queries separately, and retrieve the first 1000 results available for each intervention. We a similar query for searching Policy Commons and include documents from Working Papers, Conference Proceedings, and Reports. Khanna et al. (2021) included papers identified through literature snowballing and we will implement the same.

Since we will comprehensively search databases with a broad search query, we are likely to retrieve a large number of potentially relevant article abstracts from the bibliographic databases (~15,000 per year). To make the identification of relevant papers tractable, we will apply a ML algorithm that uses support vector machines to rank the studies identified by the search queries in the order of relevance of their abstracts. The best‐performing ML classifier will be trained on the set of previously screened documents (*N* = 6023) and iteratively trained on newly screened abstracts. While Khanna et al. (2021) used an ad‐hoc approach for deciding when to safely stop screening for additional studies, we will use a formal approach here to stop screening at the point when the probability of finding more relevant studies at a given recall level is minimal. This point is determined using a statistical stopping criterion that ensures 95% recall with statistical confidence (Callaghan & Müller‐Hansen, 2020). We extend the methodological frontier by using biased urn theory for the first time to capture the non‐random nature of documents previously screened. The stopping procedure estimates the bias with which relevant documents are screened compared to irrelevant documents because of ML prioritization. This bias is then used to estimate the chances of observing previously seen sequences of irrelevant documents—conditional on there being enough relevant documents in the as of yet unseen documents—by chance. The statistical stopping criteria would be applied to the entire pool of abstracts (~106,000) identified previously as relevant by Khanna et al. (2021) and the abstracts identified at each update. From our experience in using ML along with stopping criteria, we expect that we will need to manually screen about 10,000 abstracts. While we are aim to trigger the stopping criteria for the baseline review and yearly reviews at the 90% recall and *p* value of 0.05, higher or lower recall values could be realized with regards to resource constraints for manual screening. We will compile a comprehensive review of all the available literature yearly, following the systematic stopping criteria approach (Callaghan & Müller‐Hansen, 2020), whereas on the monthly basis we will screen at abstract level 10% of available search results and code relevant documents. The statistical stopping criteria will be adapted suitably for application to a living review and the techniques to do so will be detailed in the review methodology.

We will report study flow and selection using a PRISMA flowchart‐adapted for living systematic reviews. Campbell systematic reviews will link versions to the protocol and prior versions through its platform since this is a consideration for living reviews publication.

#### 3.3.1 Searching other resources

We will search for gray literature on RePec (title and keywords), Policy Commons (title, summar) and Google Scholar (title). For Google Scholar, we use Publish or Perish to download the relevant search results. We split the query by intervention type, implement partial queries separately, and retrieve the first 1,000 results available for each intervention. We a similar query for searching Policy Commons and include documents from Working Papers, Conference Proceedings and Reports. Khanna et al. (2021) included papers identified through literature snowballing and we will implement the same.

### 3.4 Data collection and analysis

#### 3.4.1 Description of methods used in primary research

The primary studies in our inclusion criteria compare the building energy consumption of the households before and after an intervention (pre–post), or across treatment–control groups, or both before and after intervention and across treatment groups (difference–in–difference, DID). The primary statistical methods used for analysis in these studies is difference of means, ordinary least squares regression, or panel regression with household/time fixed effects panel.

#### 3.4.2 Selection of studies

The inclusion decision will be made based on the extensive inclusion‐exclusion criteria provided in Table 3. Each study will be coded by one individual graduate student with a background in economics or psychology, so that they are trained to read the quantitative literature that is being reviewed (total number of students = 4). To achieve consistency across coders a sample of (at least five studies) will be coded by all the coders and discrepancies in the coding discussed to resolve differences.

**Table 3:** Inclusion/exclusion criteria used for classifying studies.

	Dimension	Inclusion criteria	Exclusion criteria
1.	Research question	Does the paper study the effectiveness of a behavioral, information or monetary intervention targeted at changing behavior at the household level which has energy/emissions mitigation potential?	Studies on other impacts of behavioral, information or monetary interventions apart from change in energy consumption
2.	Population	Does the paper study behavior change at the *household* level? The papers should look at household decision making and interventions that influence them. Where these decisions are measured at a higher observational level (canteen, dormitories, etc.) these can also be included.	Studies on energy consumption in industrial or commercial buildings
3.	Intervention	Does the paper study an *intervention* or *policy* targeting behavior change? This can be any intervention that falls into one of the following categories: (1) monetary interventions, (2) information interventions, (3) behavior‐based interventions, including feedback, social comparison and motivation techniques	Experiments that target specific appliances only, structural upgrades, direct load control
4.	Comparator	Does the paper use a study design which has a valid comparison group as a benchmark to quantify behavior change? This can be a control group in experimental studies, a statistically constructed or selected control group in quasi‐experimental studies or the level of behavior before the intervention in longitudinal designs.	Studies that do not observe behavior in a control group/historical reference/constructed control group or compares behavior in one treatment group to another treatment group.
5.	Outcome	Does the paper study actual behavior change that leads to change in energy consumption of the household? The primary outcome of interest is a quantitative assessment of the change in energy consumption resulting from the intervention.	Studies that investigate the shift in energy demand (e.g. whether households consume more electricity in the night when evening consumption is priced higher) but not overall reduction in energy consumption of the household are not included. Studies that only investigate intentions/motivations to reduce energy consumption are not included. Studies that only provide an effect size but not the associated variance are not included.
6.	Study type	Does the paper report on analyses of empirical data that employ experimental or quasi‐experimental (e.g., longitudinal studies, instrumental variables, difference‐in‐differences, propensity score matching, etc.)? This can include any design which either utilizes randomization or quasi‐experimental methods to establish a valid counterfactual and estimate causal effects, or a pre‐post design which utilizes longitudinal data.	Simulation, modeling or predictive studies

#### 3.4.3 Data extraction and management

The studies will be coded by two graduate students, one research associate, who have a background in economics. The risk of bias assessment will primarily be done by an experienced systematic review expert. To ensure reliability, the team will start by discussing the codebook and the interpretation of the various fields. The task will use examples given in Khanna et al. (2021). For abstract‐level coding, all the members will code a set of 50 abstracts and discuss any discrepancies. We will report Cohen's *κ* for screening at abstract level. For full text screening, all the members of the team will code a set of 10 studies that were identified to represent the diversity of study designs that we are likely to encounter and the probable issues in coding. The members will then compare the coded papers with results from other team members and discuss discrepancies.

#### 3.4.4 Assessment of risk of bias in included studies

For critical appraisal we will code for each study metrics of study quality covering aspects of internal and external validity following the risk of bias framework suggested by the Collaboration for Environmental Evidence (Collaboration for Environmental Evidence, 2022). We have adjusted the CEE framework to be applicable to the specific data set we are working with, in terms of study designs and statistical techniques implemented in the primary studies. The detailed tool is added to the codebook. The risk of bias questionnaire will be filled out by the person coding the study. To ensure uniformity across studies, 10 studies were coded by all the coders and the results compared and discussed in detail.

#### 3.4.5 Measures of treatment effect

Design elements of original studies will be captured as dummy variables for the following variables: weather controls (if a study controls for any aspect of weather, it is assigned value 1); demographic controls (if a study controls for demographic variables like age, income, composition of the family etc., it is assigned value 1); residence controls (if a study controls for the characteristics of the house like size, etc., it is assigned value 1); and randomization (assigned value 1 if households are randomly assigned between interventions). We will also include as moderator variables study design (difference‐in‐difference, control‐treatment, or pre‐post) and statistical method (panel regression, OLS regression, or difference of means tests) employed in the studies. Other moderator variables will capture the factors that are likely to affect the relationship between energy use and the treatment, for example, duration of experiment or region in which the experiment was performed.

#### 3.4.6 Unit of analysis issues

Randomization at the cluster level: we will code whether households in a given study were randomized at the cluster level (district, state, neighborhood) or at the household level. For studies, where households were cluster randomized, we will also code if the primary study calculated the effect accounting for the effect of such clustering (whether cluster standard errors were calculated). For studies where the effect of clustering was not accounted for the standard errors are likely to be artificially reduced, we will multiply the standard error of the effect estimate (from an analysis ignoring clustering) by the square root of the design effect by making suitable assumptions for the ICC. We will also perform a sensitivity analysis to check the robustness of the results of the meta‐analyses to the inclusion of the studies which do not account for clustering.

Repeated observations on participants: we will select the longest follow‐up from each study. While this may induce a lack of consistency across studies, giving rise to heterogeneity, we will also code the duration of the study to capture the heterogeneity that this introduces.

#### 3.4.7 Criteria for determination of independent findings

Generally, we do not expect the studies to capture multiple outcomes. Most of the studies included in this literature are likely to report some form of reduction in energy consumption. It could be that some studies report the reduction in energy consumption for sub‐groups of population. In this case the metric reported for the whole sample would be coded and not the reductions reported for specific sub‐groups. We explicitly exclude studies or observations that report reduction in energy consumption only for specific appliances or time of the day.

#### 3.4.8 Dealing with missing data

We will write to authors of publications to obtain the data missing from studies. This would especially be done for studies that report outcomes but not the corresponding statistical variance.

#### 3.4.9 Assessment of heterogeneity

We will examine effect size heterogeneity using by examining the results of the meta‐analysis and report the *I* ^2^ statistic for the models fitted.

#### 3.4.10 Assessment of reporting biases

We will assess publication bias using funnel plots and Egger's tests. If required, we will correct for publication bias using PET and PEESE methods.

#### 3.4.11 Data synthesis

The studies in our study are expected to report effects in terms of relative change in energy consumption but the exact dependent variable may vary across studies. We will first standardize the effects by converting the estimates of energy reduction reported by each study to semi‐partial correlation coefficients or d‐based effect sizes as appropriate and then convert them to Fisher's *Z* (Ringquist, 2013).

We will use a random effects model to aggregate the standardized Fisher's *Z* from the original studies. A random effects model is appropriate when effect sizes in primary studies do not consistently converge to a central population mean (Nelson & Kennedy, 2009; Ringquist, 2013), which is typically the case in social science research and certainly the case for studies relating to energy consumption in households with heterogeneous treatment effects (Delmas et al., 2013). We will use the most recent version of the *metafor* package in R (Viechtbauer, 2010) for implementing the random effects model restricted maximum likelihood (REML) estimator. To check that no single study exerts undue influence on the aggregate effect sizes measured, we will follow best practices and use three metrics for estimation of influence—Cook's distance, cov ratio and *τ* ^2^—from the *influence* function in *metafor* package in R. This function calculates the value of these metrics for each effect size included in the analysis.

The ordinary random effects model is inappropriate when the effect sizes included are not statistically independent (Ringquist, 2013). Effect sizes are likely to be dependent in our sample as we extract multiple effect sizes from each study. In addition, several of the studies in our set employ multiple treatments and some use data from the same underlying experiments. We will employ a multilevel meta‐analysis model to account for such dependence. The multilevel analysis explicitly models that several of the effect sizes (level 1) come from the same study (level 2). The multilevel analysis will use the default variance‐covariance structure in the *metafor* package (Viechtbauer, 2010). To test the robustness of our findings, we will employ cluster robust variance estimation using the *clubsandwich* package in R (Pustejovsky & Tipton, 2018).

#### 3.4.12 Subgroup analysis and investigation of heterogeneity

We will provide summary effect size and confidence interval in a forest plot. Effects by interventions will be estimated by including *treatment type* (monetary incentives, information, feedback, social comparison, and motivation) as interacted dummy variables in the multilevel model.

**Figure 1:**
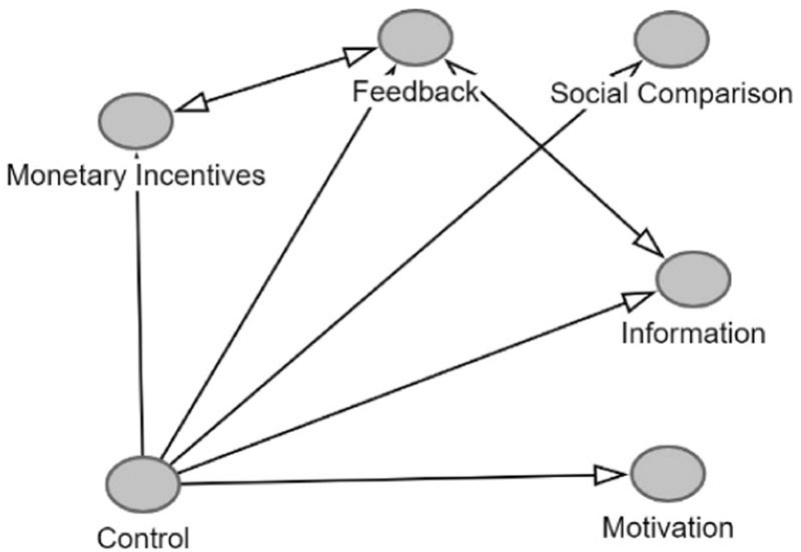
Possible comparisons in Network Meta‐Analysis.

We will also explore using network meta‐analysis (NMA) for comparison of average effect of interventions. The comparison nodes (treatments) in NMA could be monetary incentives versus control, information versus control, monetary incentives versus control, feedback versus control, social comparison versus control and direct evidence as it exists between any of the intervention categories. If the heterogeneity and transitivity assumptions can be maintained for comparisons, then the evidence across these groups can be used to provide a comparative ranking of the five major interventions (see Figure 1). Another application of NMA would be to analyze the evidence available with combination of interventions. The comparison nodes (treatments) in that case could, for example, be feedback versus control, monetary incentives versus control and feedback+monetary versus control. If the heterogeneity and transitivity assumptions can be maintained for comparisons, then the evidence across these groups can be used to compare individual interventions with their combinations.

We are guided by the Campbell (Wilson et al., 2016) and Cochrane (Chaimani et al., 2023) guidance for NMA and will publish the PRISMA flow for NMA if you decide to carry out NMA.

#### 3.4.13 Sensitivity analysis

We will conduct a sensitivity analysis of the following on results of the meta‐analysis:

- Outlier effect sizes and studies
- Publication bias
- High risk of bias studies
- Studies without a control group

#### 3.4.14 Treatment of qualitative research

The review focuses on quantitative research and we do not plan to include qualitative research.

#### 3.4.15 Summary of findings and assessment of the certainty of the evidence

We do not plan to include Summary of findings and assessment of the certainty of the evidence.

## CONTRIBUTIONS OF AUTHORS

- Content: Tarun M. Khanna, Jan C. Minx, Mark Andor, Diana Danilenko, Julian H Elliott, Max Callaghan, Tim Repke
- Systematic review methods: Jan C. Minx, Julian H Elliott, Max Callaghan, Mark Andor, Tarun M. Khanna, Diana Danilenko, Tim Repke
- Statistical analysis: Tarun M. Khanna, Mark Andor
- Information Retrieval: Diana Danilenko, Tim Repke, Luke A. Smith, Bhumika T.V., Jorge Sanchez

## DECLARATIONS OF INTEREST

The authors do not report any conflict of interest.

## PRELIMINARY TIMEFRAME

**Figure 2:**
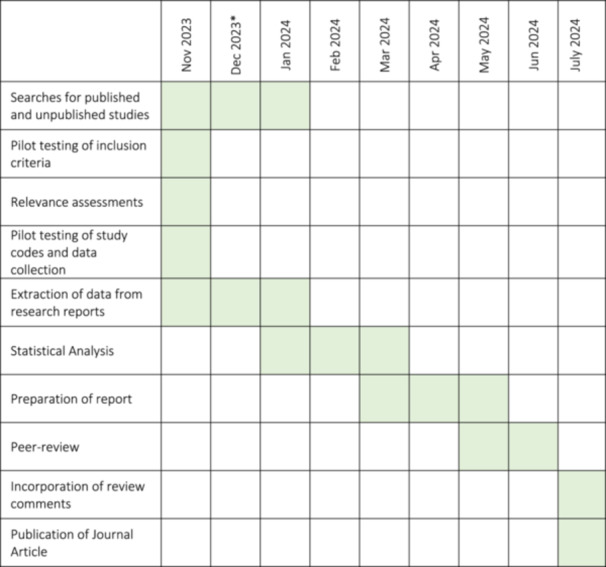
Timeframe for baseline review. *Cut‐off point for search for the baseline review. Search will continue at regular intervals for updates to the review.

The living systematic review has two parts: the baseline review that will be conducted at the start of the project and the regular updates to keep the review “living”. The timeframe for completion of the baseline review is shown in Figure 2. The baseline review is expected to be completed by July 2024.

## PLANS FOR UPDATING THIS REVIEW

**Figure 3:**
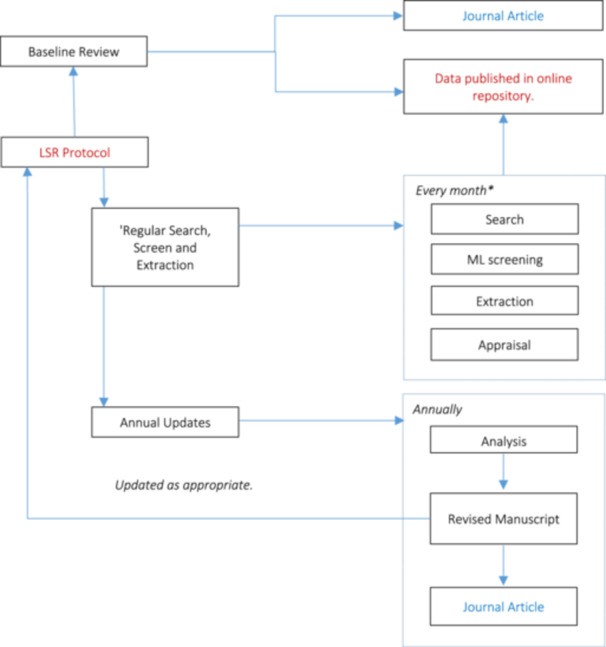
Process for updating the living systematic review. *For Scopus and Web of Science. For other databases the search and screening will be done annually.

We plan to continuously screen the new literature emerging in the field. The search queries will be run every month on Scopus and WOS, the two databases that constitute the largest source of literature for our analysis. Using the same machine learning algorithm that we used for the baseline review, we will rank order and screen at least the top 10% of all as yet unscreened documents every two weeks. Any relevant studies found will be screened and coded after critical appraisal. We will upload the data from the newly coded studies monthly on an open source platform (OSF). The appropriateness of the frequency of screening will be evaluated after the first annual update and the protocol modified accordingly. The process for updating the review is shown in Figure 3.

The search queries on the remaining databases will be run once every year. The results from these searches will be rank ordered using the machine learning algorithm. The statistical stopping criteria developed by Callaghan & Müller‐Hansen, 2020 will be applied to all search results (reranked in the order they would have been seen had all been available, including results from previous searches) until we can reject the null hypothesis that we have missed our recall target of 95% within a prespecified statistical confidence interval. At this point the screening will be stopped. The newly included studies will be coded. We plan to rerun the analysis on an annual basis. The updated analysis will feed into an updated manuscript that will be submitted to Campbell Systematic Reviews for peer‐review and a draft published as a pre‐print on the online repository.

### PEER REVIEW

The peer review history for this article is available at https://www.webofscience.com/api/gateway/wos/peer-review/10.1002/cl2.1424.

## Supporting information

Supporting information.

Supporting information.

Supporting information.

## Data Availability

n/a
