# Supplementary material for: PROTOCOL: Behavioral, information and monetary interventions to reduce energy consumption in households: A “living” systematic review
Source: Campbell Syst Rev. 2024 Jul 10;20(3):e1424. doi: 10.1002/cl2.1424 (PMC11237337; doi:10.1002/cl2.1424)
Supplement: Supplementary file 1 — Supporting information. [file CL2-20-e1424-s001.docx]

Code-book

Search Process

Meta analyses need three levels of information to be coded: search level, study level and the effect level information. We have done the first two by using the [apsis platform](https://apsis.mcc-berlin.net/scoping/) which automatically records our search strategy and study level information from the documents that have been tagged as relevant. This document mostly relates to the manual coding at the effect level, for which we used excel.

Ineligible documents

After going through the abstract and reading through the document, if you find it unacceptable to be included in the study because 1) it does not contain relevant effect sizes 2) effect size information is incomplete or irretrievable 3) document is not relevant or any other reason, please add the reason for excluding the document exclusion reason field.

Acceptable documents

- For acceptable documents we collect information on the “Effects” and the “Interventions”.
- Each study may report multiple estimates of the effect. This can be due to multiple iterations, multiple treatments/interventions, different populations, etc. You can add the multiple effects and corresponding information for each study. As such, please collect information on all the effect sizes estimated in the study along with the corresponding control variables.

Effect Fields

In this section we will capture one estimation (e.g. one coefficient, one difference of means) and the characteristics of the model used in order to accurately calculate effect sizes comparable to others included in our meta-analysis.

After the statistical information is captured it will be linked to an intervention in the next section. An effect size can be estimated for joint interventions (e.g. a TOU pricing scheme might be introduced alongside an in-home display device). The capture of the intervention section should describe the intervention linked to the estimate as accurately as possible.

| **Field Name** | **Explanation** | **Choices or Examples** |
| --- | --- | --- |
| **Document Type**  *DocumentType* | Code the type of document:  **Peer-Reviewed Paper,** if the document is an academic paper, published in a peer-reviewed journal.  **Unpublished Academic Paper,** if the document is an academic paper which has not been published in a peer-reviewed journal, e.g. a conference/working/discussion paper. | Peer-Reviewed Paper  Unpublished Academic Paper |

| Page  *Variables:*  *page* | Capture page # for the specific effect – preferably the page number that presents the table of results.  If you only have a version of the paper without page numbers use a normal page count to fill in the relevant page number. |  |
| --- | --- | --- |
| Effect number  *Variables:*  *EffectNumber* | Assign numbers to the effect making sure they are identifiable at a later stage. In general, numbers should get larger for effects which appear later in the text. | Attention: effect numbers do not always start with a 1. There is no particular meaning to the effect numbers and missing numbers do not imply missing effect sizes. |
| Statistical technique  *Variables:*  *statistical_technique* | Studies may employ different techniques to estimate effects.  Note: Don’t code Difference in Difference as effect statistical_technique. Here we really just capture the type of regression and let Difference in Difference be the study type |  |
| Dependent variable  *Variables:*  *dependent_variable* | Studies will utilize various measures to capture energy consumption. What variable/operationalization are they using here. We are ultimately interested in percentage change in energy consumption of the household given the intervention.  Note.  We still capture experiments where households own automated (smart) thermostats, as long as the settings of the thermostat can be overridden at any time (eg. Herter (2013))  We always capture the ITT and disregard ATT/LATE estimates.  Write the dependent variable and the unit between brackets | Example: household hourly electricity consumption (kWh) |
| Study design  *Variables:*  *study_design* | Whether the study calculates a pre-test/ post-test effect size or a control-treatment effect size  *Pre-test/ post-test*- No control used, same group measured twice  *Control-treatment* - no baseline, two groups with starting observation started at the same time  *Difference in Difference* - baseline for both control and treatment groups followed by treatment and measure of effect |  |
| ^­^Effect size - statistical estimate (coefficient)  *Variables:*  *coefficient* | Capture the *absolute* value of the relevant regression coefficient. |  |
| Baseline consumption  *Variable:*  *Baseline Consumption*  Notes_baselineConsumption | Record the average consumption in energy units (eg. kW/h) during the period which the coefficient/difference in means refers to.  We want use this variable to calculate the percentage change in energy consumption during the analyzed period when the coefficient/ difference in means must be interpreted as the change in energy units (dividing the coefficient/ difference in mean by the baseline consumption).  Rules for recording the baseline consumption:  **Which consumption?** If baseline data was collected and average consumption during that time is reported, record that. If baseline is not available, record the average consumption during the experiment.  **Whose consumption?** If average consumption of all households is mentioned, record that. If not, then the weighted average consumption of treatment and control group should be reported. If both the options are not possible, report the average consumption of the control group.  Preference order:   1. Average consumption of all households (T+C) during baseline period 2. Weighted average consumption of T and C during baseline period 3. Average consumption of all households (T+C) during the treatment period 4. Weighted average consumption of T and C during treatment period 5. Consumption of the control group |  |
| Direction of effect size  *Variables:*  *effect_direction* | Capture the direction of the effect of the intervention. | NA  Increase  Decrease  No change |
| Coefficient SD  *Variables:*  *coefficient_sd* | The variance is captured by the standard error of the coefficient.  If a standard error is not provided use code -999 and use ‘not provided’ as the type |  |
| Coefficient SD type  *Variables:*  *coefficient_sd_type* | Also capture the type of uncertainty measure provided (see right). |  |
| Missing Uncertainty measures :  *Variable :*  *uncertaintyMeasuresMissing* | Indicate if uncertainty measures are missing but sample size is provided.  Leave blank if uncertainty measures are provided. | No uncertainty measures only sample size available |
| Significance  *Variables:*  *p_value*  *significance_bound*  *significance_test*  *test_statistic*  *effect test_tails* | Also capture the t-statistic.  If a study provides the coefficient and standard error, calculate the specific t-statistic (β/se) and use that to calculate a corresponding P-value.  If a study only provides the level of significance (no standard error, no t-statistic, no specific p-value) then capture the level of significance in the p-value field and calculate the t-statistic using the level of significance and mark it as a lower bound (if significant) or choose the p-value 0.1 and upper bound (if not significant)  [**https://handbook-5-1.cochrane.org/chapter_7/7_7_3_3_obtaining_standard_deviations_from_standard_errors.htm**](https://handbook-5-1.cochrane.org/chapter_7/7_7_3_3_obtaining_standard_deviations_from_standard_errors.htm) | *For significance bound, the key for coding in the BI review was*  *1 - lower bound*  *2 - upper bound*  *3 – actual*  *Check and redefine such that the variable takes on one of the following values*  *NA*  *lower bound*  *upper bound*  *actual* |
| Effect size - difference of means  *Variables:*  *control_mean*  *control_sd*  *treated_mean*  *treated_sd*  *diff_mean*  pooled_sd | Capture both the control and treatment group means where possible. Otherwise capture the difference along with the value of the relevant test statistic (t, Chi or F).  Calculate pooled standard deviation using the formula in Ringquist if required. | If the control sample size is not clearly specified, take a conservative approach and use the treatment sample size as total sample size. |
| Sample Size  *Variables:*  *control_sample_size*  treatment_sample_size  total_sample_size | Capture as much detail as possible (i.e. if all three options are given, record all options indicated below). Given that a specification can include multiple treatments, the sum of treatment and control sample sizes will not necessarily add to the total.  Studies may run analysis comparing treatment groups only to the control, or also to each other, capture elements    **Total** – Control and all relevant treatments, full sample size (for pre-and post- treatment set-ups)  **Treatment** – Capture sample size for specific effect being captured (this should correspond to one treatment or combination of treatments)  **Control** – There is possibly only one control group for multiple treatments |  |
| Control Definition  *Variables:*  *controls Electricity use*  *controls Energy prices*  *controls Environmental attitudes*  *controls HH controls (demographics)*  *controls None - base model*  *controls Residence controls*  *controls Seasonal controls*  *controls Weather controls* | List the controls which are being used to better isolate the effect of the intervention. Besides the variable of interest (intervention) which elements that affect energy consumption are included.    Fixed effects and random effects for households or time effects should NOT be captured here. These should be captured in the statistical technique. Only include explicit weather or seasonal controls in the analysis.  If in pre/post analysis the baseline is corrected for weather, code 1 for weather controls. | **Weather controls** (heating degree days or cooling degree days, etc.)  **Seasonal controls** (monthly dummies or quarterly/seasonal dummies, etc)  **Energy prices**  **Residence controls** (physical nature of the house, size of house, appliance stock, etc.)  **Household controls** – demographic info (income, age, number of residents, education, etc.)  **Base energy consumption** |
| Geography and Aggregation Level  *Variables:*  *geographic_scope*  geographic_location  *Notes: Study scope*  *City*  *State*  *country* | **Geographic scope** captures the area across which the intervention was carried out  Note: Try and stick to the options mentioned  Leave a note if smaller than municipality.  The field **Geographic location** should not be coded anymore. Instead, use the separate fields city, state, country.  Note: Try and stick to the format city, state, country | For Geographic scope:  smaller than municipality, municipality, state, town, county |
| Smart metering  *Variable:*  *smart_metering* | If smart metering was used at which aggregation level was the energy consumption metered.  Choose “not specified” if smart metering was used but the aggregation level is not reported.  Do not fill out. | Yes/No  NA |
| Randomization dummy  *Variable:*  *randomisation_dummy* | Were households randomly assigned to control and treatment group and/or between different treatment groups? | Yes/No |
| Opt-in vs. Opt-outs  Variable:  Opt_in | Were households first selected and then allowed to opt out of the intervention or were the households required to opt-in to the intervention or neither  Note: Write “Opt in” when households are given the option to opt-into the experiment explicitly. Choose “Opt out” when households are given an option to drop out of the experiment explicitly.  Choose “Mandatory” if the participation in the scheme was mandatory with no option to opt-out. | Opt in  Opt out  Mandatory  Neither  Unclear |

Intervention

| **Field Name** | **Explanation** | **Choices or Examples** |
| --- | --- | --- |
| Out of sample bias  *Variable:*  *outOfSampleBias* | Were the control and treatment group not representative of the average population of the corresponding area?  Choose probably yes if the sample is not representative and there probably is an out of sample bias. If participants can self-select into treatment choose probably yes.  Choose probably no if the authors convincingly argue and show that their sample is representative. | Probably Yes  Probably No  Unclear |
| Reporting biases  *Variable:*  *reportingBiases* | Was there bias as to which effect sizes (outcome reporting bias) or which estimates (analysis reporting bias) are reported by the authors?  If the paper manages to convince you that they have done a good job, choose probably no. | Probably Yes  Probably No  Unclear |
| Total Duration  *Variable:*  *interventionDuration* | Capture the *total* duration of the experiment.  Note: Data for duration should be in weeks where 1 month = 4 weeks and 1 year = 52 weeks | Pre/post setting: experiment runs for 3 months, control period is the same 3 months in the year before -> 12  An experiment runs for two subsequent summers. The intervention duration in the first summer is 12 weeks, in the second summer the intervention duration is 64 weeks.  interventionDuration is the total duration of the study including the baseline observation period, treatment period and follow up period if any. |
| Treatment Duration  *Variable: InterventionTreatmentPeriod* | For how long did the *treatment* of the intervention take place.  Note: Data for duration should be in weeks where 1 month = 4 weeks and 1 year = 52 weeks | If two separate periods are pooled together, the intervention duration is the sum of both intervention treatment periods. |
| Follow-up dummy  *Variable:*  *interventionFollowup* | Record yes if a follow-up investigation into energy consumption was conducted and no otherwise. | Yes  No  Unclear |
| Framing Unit  *Variable:*  *framing_unit* | When participants are given information or feedback on their energy consumption, in what units/terms is this information given | Energy (kwh)  savings ($)  Co2  other efficiency (R-value)  other ecological |
| Timing/Frequency  *Variable:*  *informationFrequency* | What is the frequency at which the households are contacted to provide feedback/ information? Normally not applicable for pricing interventions. | Continuous (on-demand)  Monthly  Bi-weekly  One time only  Monthly or quarterly |
| Medium  *Variable:*  *medium* | How was the household contacted? Could be relevant for all types of interventions. |  |
| Intervention types and sub-types | We look at five broad intervention types: Information, Feedback, Monetary Incentives, Social Comparison and Motivation | Information: home audits, tips,  Feedback: historical, enhanced billing, in-home display  Social comparison: peer comparison, HER  Monetary Incentives: Rewards, ToU, Real-time Pricing, Dynamic Pricing, Peak Load Pricing  Motivation: Gamification, commitments, goal setting |
| Exclude effect size  *Variables:*  *EffectSizeExclusion*  *EffectSizeExlusionReason* | If an effect size was already coded, but you thereafter realize that it can not be included e.g. because of missing uncertainty measures choose “exclude” and describe the reason which lead to this decision. | *Exclude* |
