# Supplementary material for: PROTOCOL: Behavioral, information and monetary interventions to reduce energy consumption in households: A “living” systematic review
Source: Campbell Syst Rev. 2024 Jul 10;20(3):e1424. doi: 10.1002/cl2.1424 (PMC11237337; doi:10.1002/cl2.1424)
Supplement: Supplementary file 2 — Supporting information. [file CL2-20-e1424-s002.docx]

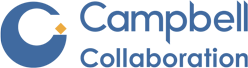


PEER REVIEW FEEDBACK CAMPBELL COLLABORATION

**Title of protocol:** Role of behavioral and monetary interventions in reducing energy consumption in households: A ‘living’ systematic review

**ID:** CSCG-23-02/cl2.20230152

|  | **Peer Review comments** | **Editorial Comments** | **Author’s response** |
| --- | --- | --- | --- |
| **GENERAL POINTS**  Campbell accepts non-intervention reviews such as reviews of outcome measures, risk/protective factors, methods, qualitative evidence and overviews or review of reviews.  The review should be written in  language that is easily understood. |  |  |  |
| **TITLE**  (Only comment if you strongly disagree with the current title) | **Methods editor:** This refers to behavioural and monetary interventions, but the abstract also mentions information. If these are considered a subset of either behavioural and/or monetary interventions, that could be clearer in the abstract and text. In addition, I wondered whether you also include regulation (e.g., enactment of laws or monitoring compliance against laws) and if not why not? | **Agree-please describe whether information is a component of behavioural or monetary in the abstract and throughout the text** | Title edited to include information. The fact that information incentives are included and categorized separately is mentioned in the introduction and Table 2. We will not include any regulation such as mandatory increases in energy efficiency standards. Regulation that requires use of behavioral, information or monetary incentives like Home Energy Reports or Time of Use pricing are included when their effects are studied as a part of a study. |

|  | **Abstract** (please note these points also apply to the main text)  Please ensure that all relevant monetary and behavioural intervention areas that you intend to cover are captured using appropriate language. Preferably the discussion of policy actions will be comprehensive and mutually exclusive. | **I believe table 2 covers these concepts** | We have moved the table with definition of interventions inline to improve readability. |
| --- | --- | --- | --- |
|  | Would it be clearer for the non-specialist reader to say “engineering” rather than “power system studies” (this point also applies in the background section para 2)? | **Consider at your discretion** |  |
|  | The review questions should be worded more neutrally: “to what extent” rather than “by how much” (which allows for interventions to now work, or even be harmful); “what is the relative effectiveness of interventions” rather than “which interventions are most effective” (allowing for discussion of which interventions are less effective than others). Should “under what conditions” be a separate question? Should there be an additional question on equity implications of these interventions, particularly for the poor? | **100% agree that Campbell reviews should be worded neutrally since we aim to provide a balanced assessment of the evidence. Please address** | Text has been edited to make it neutral. Equity implications of interventions are out of scope and need to be addressed in a separate review. The studies that investigate equity implications of interventions are distinct and capture energy consumption at a much more granular level than those included in our dataset. |
| **BACKGROUND**  The background should present the problem, issue or condition being addressed, the description of the intervention and justification for why an EGM is needed in light of policy, practice priorities and existing or prior EGMs or reviews | **Methods editor:** There is lots of focus in the first paragraph on lack of systematic knowledge about climate solutions in general, whereas I suspect systematic reviews have appeared since the Berrang-Ford and Minx papers cited. Since the topic of this review is specifically on energy consumption by households, can the text be modified to talk about 1) what role energy consumption by households is thought to play in overall climate change mitigation efforts, and 2) the limited systematic knowledge on what works to reduce household energy consumption specifically.  Does the background need to consider a bit more carefully that this is a partial assessment and notably excludes interventions on the supply side of the market? I think so, partly because of the issue we know about of fossil fuel greenwashing, where the onus on action is placed on the consumer and not the producer. | **At your discretion to address; please justify your response**  **Please respond as to whether you agree** | We will highlight the need for a review that captures the role of energy consumption in households. However, we emphasize that the review seeks to set a culture of living reviews for climate policy in general and not just address gap in systematic evidence on household energy consumption.  Demand and supply policies are clearly recognized as being distinct and equally necessary for climate mitigation. We have added citation to the relevant literature to highlight the importance of demand side, even outside of green washing. |
|  |  |  |  |

|  | In the final paragraph, please be more hesitant when speaking about being the “first” to do things. It reads better to say what contribution the review will make to expanding methods, standards and research, rather than insisting that you will be the first to do these things. But see also the suggestion to move this paragraph under objectives. | **Agree that there is no need to call this the “first of a kind” particularly since there are many other living reviews. I agree this could be**  **framed as an objective** | Comment noted and text modified accordingly. |
| --- | --- | --- | --- |
| **OBJECTIVES**  This section should present a concise set of statements that identify the objectives of the review. | **Methods editor:** This text repeats text in the background section (and is now the first time I am reading about “power system studies”); please reduce this repetition.  I note that the final paragraph of the background gives some methodological objectives for the review, which I would include in this section. Note also the points made above about ensuring review questions are neutrally worded. | **I suggest to only include the objective here, and move the first two sentences of objectives to the background if they are needed**  **Agree as per above** | Comment noted and text modified accordingly. |
| **CRITERIA FOR CONSIDERING STUDIES**  Eligibility criteria are described. Examples include study design, population, intervention, comparator, outcomes. | **Methods editor:**  Criteria for inclusion/exclusion: please ensure that the classification of interventions given here is used elsewhere in the protocol, and ensure that the text summarises key aspects of the PICOS which are given in the table. In the text/table, please clarify:   - populations, whether global or if restricted to particular regions; and clarify that public places like hospitals and schools are excluded. - interventions which are excluded – for example, it is not clear whether regulations are included or not. - comparators means what the comparison condition is – whether no intervention, business-as-usual, an alternative | **Agree that this section should cover PICOS. I suggest requesting that Table 2 is inserted inline with the text when this is copy-editted** | The relevant table is inserted inline with the text under the heading “Criteria for considering studies for this review” as per the new template. |

|  | intervention, or a wait-list (pipeline study). What you have presently included under comparator would be more appropriate for study design.   - If electricity consumption is the primary outcome which must be reported for the study to be included, specify this. If other outcomes are secondary (and hence only reported if a primary electricity consumption outcome is reported), specify this, and whether they are limited to behaviours or also knowledge, attitudes and wellbeing (quality of life) outcomes.   -   - Some of the exclusion criteria do not sound reasonable: e.g., “Studies in which sample size is too small (<10) to extract meaningful estimates” – sample size is not a condition for exclusion from a Campbell review (sample size can be assessed using risk of bias, and meta-analysis is used to address issues of statistical power in primary studies); - ​ - ​ - ​ - “Studies that only provide an effect size but not the associated variance are not included” – this is fine for excluding the study from the meta-analysis (but note that methods can be used to re-engineer variances from other information that is reported), but not for exclusion from the systematic review itself. - “Studies that only investigate   intentions/motivations to reduce energy consumption are not included” – yes but are these eligible as secondary outcomes?   - ​ - “Studies that investigate the shift in energy demand (e.g. within a day) but not overall change” – I don’t understand this point, so it would be useful to give an example. | **It is mandatory to specify the role of outcomes in Campbell reviews and whether they will be used as inclusion or exclusion criteria.**  **Agree that sample size is not usually used to exclude studies; it would be better to exclude on the basis that the studies do not meet your design criteria**  **Campbell specifically does not exclude studies due to lack of variance data. Rather, these studies should be described as included studies, with a note that no variance could be extracted. Please respond to this point** | Electricity consumption is the primary outcome reported in the studies and is usually the only outcome as well in the studied literature. Inclusion of other outcomes will lead to exclusion of many studies.  We will include such studies if recommended. However, it is to be noted that the studies in question do not even report averages but rather only individual outcomes for 4-5 houses. If possible, averages will be calculated, and studies included.  We clarify that we try and estimate the standard deviation associated with the outcome variable in all possible ways possible (back-translating from information that is reported e.g. t-stats, p-values, confidence intervals etc.). But if none of this information is available regarding the outcome standard deviation, then we exclude it from our analysis.  Since we cannot use these studies for our analysis, we would not include them in the review but can include a list of papers that were excluded based on this criteria and include it in the supplementary material so that the studies are recorded and available for future research.  Points regarding demand shift have been clarified in the protocol. |
| --- | --- | --- | --- |

|  | - Study design: experimental, quasi-experimental and longitudinal designs: it is not sufficient to say “quasi-  experimental”, we need to know what types of designs (Figure 2 in this paper gives an example of how to categorise designs [https://www.tandfonline.com/doi/full/10.1080/19439342.201](https://www.tandfonline.com/doi/full/10.1080/19439342.2012.711765) [2.711765](https://www.tandfonline.com/doi/full/10.1080/19439342.2012.711765)). Also please note that longitudinal studies that aim to estimate a causal effect of a non-randomised intervention on an outcome are by definition “quasi-experiments”. | **Please respond to this** | Apologies for the typo. Longitudinal studies are considered quasi experimental. Other study designs that estimate a causal effect including difference-in-difference, IV, etc. are included in quasi experimental designs. This has been updated in the protocol. |
| --- | --- | --- | --- |
|  | It would be useful to present a theory of change for the intervention(s) that articulates the types of interventions, intermediate outcomes and final outcomes. The text around this should discuss the underlying assumptions in the ToC. | **Theory of change is desirable but not mandatory** |  |
|  | **Methods editor:** You should incorporate other literature sources like | **Please respond** | We include literature snowballing in our search. Please see response to others below.  The studies will be coded by two graduate students, one research associate, who have a background in economics. The risk of bias assessment will primarily be done by an experienced systematic review expert. To ensure reliability, the team will start by discussing the codebook and the interpretation of the various fields. The task will use examples given in Khanna et al. (2021). For abstract level coding, all the members will code a set of 50 abstracts and discuss any discrepancies. We will report Cohen’s Kappa for screening at abstract level.  For full text screening, all the members of the team will code a set of 10 studies that were identified to represent the diversity of study designs that we are likely to encounter and the probable issues in coding. The members will then compare the coded papers with results from other team members and discuss discrepancies.  To ensure greater consistency and correct for possible errors, a random sample of papers will be drawn and cross checked. (approx. 10)  The protocol has been updated with this information. |
| **SEARCH METHODS AND SOURCES** | working papers and dissertations, and literature snowballing (forwards |  |  |
| Search strategy is comprehensive | and backwards citation tracing). Open Alex is now available to help |  |  |
|  | If you are only double-coding a proportion of studies, you should | **Please respond to this;** |  |
|  | report inter-rater reliability metrics (e.g., Cohen’s Kappa). I would also | **The Campbell MECCIR** |  |
|  | question whether a random sample of 50 abstracts and 5 full texts is | **standard is that it is** |  |
|  | sufficient if you are using the decisions to train the machine learning | **highly desirable to use 2** |  |
|  | searches. | **coders, and if you do not** |  |
|  |  | **it recommends:** Where  large number of studies makes this procedure too demanding, random samples of the studies can be drawn and recoded by a different team member so that the  reliability of the coding can be assessed and reported. The procedures planned for training coders and checking their accuracy before they begin providing data for the review should also be described along with the relevant background of those expected to do the coding. |  |

|  | **Information Specialist:**  **Sources**  Scholarly literature: The databases Web of Science Core Collection, Scopus, and JSTOR (via Constelate), are appropriate. However, the research team may want to consider including some disciplinary databases in Business and Environmental Sciences, such as Business Source Complete and Environment Index (both on the EBSCOhost platform). These databases are smaller but more discipline-focused. They could be searched on the larger time interval (annually).  Grey literature: The protocol doesn't mention anything about searching for grey literature or include any grey literature sources. The author team might consider sources such as SSRN, Policy Commons, among others. I ran a quick search in both the above mentioned grey literature sources and relevant studies seemed present. Given that the interventions of interest are expected to be conducted and published by government and industry, including a few grey literature sources would enhance comprehensiveness. MECCIR (C12 and C28) encourages authors to consider relevant grey literature.  **Search Strategies**  Outcome concept:   1. Consider adding the words (power OR heating) alongside energy OR electric OR electricity in the proximity search string. 2. Consider shortening the proximity length. 15 seems large and   could be reduced to improve the noise ratio. I would recommend somewhere in the range of 5-7. | As per above comment about REPEC, please justify why your search is sufficient if you do not think these databases are needed  Searching grey literature is mandatory in Campbell; please respond as to whether your search will encompass any grey lit, and justify if not  Please consider and respond. If you do not think these are necessary, please justify. | As per the guidance given by the editor, we have decided to include REPEC and Policy Commons while searching for literature. The review protocol has been updated. We use a simplified query allowed by the database for searching Policy Commons, including documents from Working Papers, Conference Proceedings and Reports.  Adding add “OR power OR heating” to the query returns an additional ~ 6000 abstracts. We ran our machine learning model to test the relevance of these additional abstracts. Only 29 had predicted relevance value >0 (0.1 and 0.2). We also reviewed the first prioritized 100 abstracts and didn't find any relevant documents.  Since we are using ML to prioritize documents, we do not consider necessary to reduce the number of abstracts that we have at this stage. |
| --- | --- | --- | --- |

|  | 1. The term "price responsiveness" should be moved to the intervention concept as it seems out of place here, and does not represent the "energy" aspect of this concept.   Population concept:   - 1. Consider including "prosumer*" as a keyword   Intervention concept:   - - 1. Consider including the following terms or phrases: "rate design" OR "demand response program*"   Transparency and replicability of the search strings:  While the terms including Boolean, truncation and proximity operators have been provided, there is currently no information on which search fields the authors will use in each of these databases. Without this, the search strategy does not meet the MECCIR transparency requirement.   - - - - In Web of Science core collection, I suggest using the Topic field for each concept/line       - In Scopus, I suggest the default which is Title-Abstract- Keywords.       - I do not have access to the JSTOR instance the team is using, so cannot propose search fields for this source.   Google Scholar: How will the records be exported from the Google Scholar search, since it does not have its own batch exporting mechanism? Do they plan to use a scraping program like Publish or Perish? Furthermore, the current search string provided leads to a large number of results, and Google Scholar has a 1000 record limit.  i.e. you cannot scroll past 1000 records even if you plan to use a scraping program to export the records page by page. Even limiting to a single year (e.g. 2023) results in more than 1000 records. The authors will need to re-consider their Google Scholar search string to work within the constraints of the search system. | Optional-please respond.  **Please respond, with justification.**  **This is a requirement; please add these details**  **Please respond as to whether these were considered, with justification.**  **Describe how this will be handled** | We are excluding studies where the household is a prosumer (see exclusion criteria). Monitoring and dynamics of energy consumption are different and more complicated than those addressed by other studies.  We do not include papers that simply look at rate designs for electricity. These designs are not intended to reduce consumption but rather enhance the economics of the utility/ consumer and therefore are not comparable to other interventions. Similarly demand response program is a generic term that also includes deployment of automatic load control technology that we do not intend to study here.  We also conducted a check by incorporating all the suggested changes in the query together. Overall, including all the suggested changes results in only marginally more abstracts (~1000) which is comparable to the original query. Seeing that the work has already been done and the changes would be marginal, we would prefer to keep the original query.  We search the databases at the Title-Abstract-Keyword level where possible. However, the fields vary somewhat by databases. The information is contained in the revised protocol.  For Google Scholar, we use Publish or Perish to download the relevant search results. We split the query by intervention type, implement partial queries separately, and retrieve the first 1,000 results available for each intervention type. |
| --- | --- | --- | --- |

|  | Search updates and data management:  The details regarding how the searches will be re-run and how the data will be managed is insufficient and therefore not clear.  For example, will the biweekly WoS and Scopus searches be done via alerts or using search commands? Currently, Web of Science only offers daily, weekly, or monthly email alerts. If using search commands, how will the team limit the searches to a 2-week period?. Or is the team planning to re-run and export the entire search and deduplicate against the previous searches every two weeks? Based on feasibility, the team might consider updating the Web of Science and Scopus searches every month rather than every two weeks. Despite the choice of update frequency, more detail needs to be provided on how the searches will be updated. | **In addition, I found the description in the tables that searches would be run annually and updated annually seemed to contradict the text which described searching every 2 weeks. In living reviews, it is typical to describe how often the searches will be done, how often the results will be screened, and if the results of screening will be provided in an updated repository of relevant studies somewhere. Campbell does not have the ability to update the record every 2 weeks, but you could use an open access repository such as OSF or Figshare for this, and it would document versions of the list** | As noted by the information specialist, we cannot limit a search to two weeks in WoS and Scopus. As advised by the information specialist, we will search these databases monthly instead.  *The remaining* databases will be searched annually. This was clarified in the text in the review protocol but was not clearly specified in the figure, which unfortunately caused some confusion. The figure has now been revised.  The studies identified and coded will be uploaded on OSF every month. We will re-run the complete analysis and update the manuscript annually. |
| --- | --- | --- | --- |

|  | How will the search results be deduplicated? Using software? If so, which software will be used.  Supplementary searching:  Do the authors plan to search the reference list of every included study. This is mandatory according to MECCIR C30.  For searching the grey literature, and ensuring proper records and data management, and reporting, the team might want to consult or work with a librarian. | **Please respond**  **Even though this is mandatory, I am not sure this is possible for 300 references, unless by automating this task.**  **Please consider adding this if you are doing this, or justifying why not, if not.**  **Does the team have a librarian? It would be good to mention this in acknowledgements if so.**  Will you report details on how you judge whether the stopping criteria is met, and any associated data? | We have developed our own platform (NACSOS) to manage the studies identified in searches. This platform has a deduplication feature built into it.  Most of our data comes from Khanna et al (2021), which did literature snowballing. Khanna et al (2021) found that only limited papers were identified through snowballing that were not already identified by other sources. So, we do not plan to conduct snowballing for the papers identified in 2024.  We do not have a librarian on the team.  Yes, we will report the statistical measures for the stopping criteria and provide the associated analysis. |
| --- | --- | --- | --- |

| **METHODS**  Campbell reviews are reviewed by a statistical and methodological expert, so we ask referees to only comment on aspects of methods which relate to their content expertise such as defining eligibility criteria, categories for subgroup analysis or moderator analysis, or specific keywords to use in searches. Campbell reviews should be based on explicit, transparent, and reproducible methods and procedures. Therefore, the methods should be presented in sufficient detail to allow a knowledgeable reader to assess the quality and appropriateness of the plan for conducting the review and, if desired, to reproduce the main features and findings of the subsequent review by following that plan. | **Methods editor:** Para 1: What is “this study-in-methods”? I thought this was a living review of intervention effects, not just a review of methods.  **Independent findings**  Whether or not you expect it, if a study reports, say, knowledge outcomes alongside energy consumption, will you report and meta- analyse these separately? What will you do about studies that report multiple regression specifications - will you collect effect sizes for all and calculate a “synthetic mean”, or choose the one with lowest risk of bias? Finally, what about multiple reports of the same study?  **Study coding**  Aloe and Thompson recommend using the semi partial correlation coefficient effect size for adjusted studies like the ones you are using. 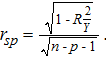 where 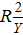 is the squared multiple correlation for the regression model (equal to the coefficient of determination for a linear model including constant), and n-p-1 the degrees of freedom. [Aloe, A.M. and Thompson, C.G. 2013, “The Synthesis of Partial Effect Sizes”, Journal of the Society for Social Work and Research, 31 December, vol. 4, no. 4, pp. 390–  405. <https://doi.org/10.5243/jsswr.2013.24>] You are not required to use it, but it would be useful to understand the properties of the effect size metric you are proposing to use in light of other metrics like this. | **I found this term “study- in-methods” out off place also since the main focus is to assess effectiveness of these interventions. I suggest to remove and simply reference living review guidance**  **Please address these comments. For multiple reports of the same study, these should be analyzed as one study with multiple publications, and you need to decide which study will be used for analysis and how you will decide.**  **Consider adding details on the effect size metric you are using** | Removed as required  We do not plan to code information on knowledge outcomes alongside energy consumption.  For reports that use the same underlying study, we will treat them as one study with different outcomes reported. We will collect all the effect sizes available from all reports. We use a multi-level model to account for the possible interdependence between various reported effect sizes.  We will standardize the effect sizes to semi-partial correlation coefficients or d-based effect sizes as appropriate. Given the limitations of these metrics (See Meta-analyses of partial correlations are biased: Detection and solutions  T D Stanley 1 2, Hristos Doucouliagos 1, Tomas Havranek <https://pubmed.ncbi.nlm.nih.gov/38342768/>), we will convert these into Fisher’s Z score and use that for the analysis. |
| --- | --- | --- | --- |

|  | **Statistical procedures**  There should be a commitment to present forest plots that transparently demonstrate effect size heterogeneity and central tendency. There should also be a discussion of tests for small-study effects/publication bias.  If you are to answer the question about comparative effectiveness, you will need to use network meta-analysis, since other methods do not adequately capture the full range of interventions and are likely to be underpowered. It is also important to consider the comparison conditions in interpreting relative effect sizes (see comment above), for which data should be collected. | **It was not clear to me if you would present forest plots. Can you clarify please?**  **I agree with this and see you mention that NMA will be explored, but you do not give criteria for how you will decide whether to use NMA, and if you do use NMA, you will need to report on whether the assumptions are reasonable (eg Transitivity assumption, coherence?). I suggest you could keep this brief by referring to the Campbell policy brief on** [**NMA by David Wilson**](https://onlinelibrary.wiley.com/doi/10.4073/cmpn.2016.1)**.** | Given the large number of studies (>150) and effect sizes (>400), we do not believe meaningful forest plots can be generated.  We have consulted the Campbell policy brief to explore application of NMA to this use case. We are indeed studying where the assumptions for applying NMA are reasonable for the data that we will collect. This would be the basis for deciding whether NMA can be used or not. If any more guidance can be provided on application of NMA, we would definitely welcome it. |
| --- | --- | --- | --- |

|  |  | For critical appraisal, you cite the Cochrane risk of bias2 tool, but you state  the “Campbell Systematic Reviews tools”- this section is not clear to me.  I see that you state “modified RoB” in the MECCIR-can you please add more detail on the tool and how it will be applied to the different types of designs that are eligible?  You do not say if you will have 2 people screening for eligibility (highly desirable but not mandatory), and you do not say how many people will code risk of bias. It is ok if just one but it is mandatory to report this. Please add  Will you assess publication bias? This is highly desirable in our MECCIR, and might be especially interesting given you are using a stopping criteria and machine learning to identify studies | We have adjusted the CEE framework to be applicable to the specific dataset we are working with, in terms of study designs and statistical techniques implemented in the primary studies. The detailed tool is added to the codebook.  The risk of bias questionnaire will be filled out by the person coding the study. To ensure uniformity across studies, 10 studies are coded by all the coders and the results compared and discussed in detail.  We will assess publication bias using funnel plots, Egger’s tests and try to correct for publication bias using PET and PEESE methods. |
| --- | --- | --- | --- |

| **ADDITIONAL COMMENTS ON THE PROTOCOL IN GENERAL**  Are there other issues that should be taken into account in the conduct of this review? | **Methods editor:** Please ensure a final read and spelling/grammar checks are done before submission due to typos: e.g. “how scientific evidence on the effectiveness of one particular set of policy interventions can, namely behavioral, information and monetary interventions in household energy demand, can be...” (p.2)  “Jeremy Grimshaw” not “Jeremey Grimshaw” | 1. I suggest to add that you will report study flow and selection using a PRISMA flowchart-this is mandatory. There is also an [article describing](https://www.ncbi.nlm.nih.gov/pmc/articles/PMC8804909/) [reporting for flow](https://www.ncbi.nlm.nih.gov/pmc/articles/PMC8804909/) [for living reviews](https://www.ncbi.nlm.nih.gov/pmc/articles/PMC8804909/) which may be helpful. 2. There is a PRISMA for living systematic reviews underway; Protocol is here ([https://f1000res](https://f1000research.com/articles/11-109) [earch.com/article](https://f1000research.com/articles/11-109) [s/11-109](https://f1000research.com/articles/11-109)) . I   suggest reporting according to this once it is available.   1. I suggest you mention that Campbell systematic reviews will link   the updated | Noted and the protocol has been updated accordingly.  Noted and the protocol has been updated accordingly.  Noted and the protocol has been updated accordingly. |
| --- | --- | --- | --- |

|  |  | versions to the protocol and prior versions through its platform since this is a consideration for living reviews publication  4) Will there be a stopping criteria for updating this review? How will it be decided? This is a common feature of living systematic reviews- eg [https://systemati](https://systematicreviewsjournal.biomedcentral.com/articles/10.1186/s13643-023-02325-y) [creviewsjournal.bi](https://systematicreviewsjournal.biomedcentral.com/articles/10.1186/s13643-023-02325-y) [omedcentral.com](https://systematicreviewsjournal.biomedcentral.com/articles/10.1186/s13643-023-02325-y)  [/articles/10.1186/](https://systematicreviewsjournal.biomedcentral.com/articles/10.1186/s13643-023-02325-y) [s13643-023-](https://systematicreviewsjournal.biomedcentral.com/articles/10.1186/s13643-023-02325-y) [02325-y](https://systematicreviewsjournal.biomedcentral.com/articles/10.1186/s13643-023-02325-y) | Yes, we will use the stopping criteria to update the review on an annual basis. While we search the two largest databases every month, we will consolidate all searches across all the databases at the end of the year and check if more abstracts need to be screened to meet the stopping criteria at the time of the annual update. |
| --- | --- | --- | --- |
